# Nucleotide metabolic mismatches in mammalian hearts: implications for transplantation

**DOI:** 10.1308/003588413X13511609955571

**Published:** 2013-01

**Authors:** Z Khalpey, MH Yacoub, RT Smolenski

**Affiliations:** ^1^University of Arizona,US; ^2^Imperial College London,UK; ^3^Medical University of Gdansk,Poland

**Keywords:** Ecto-5’-nucleotidase, Adenosine, Xenotransplantation

## Abstract

**Introduction:**

Human donor organ shortages have led surgeons and scientists to explore the use of animals as alternative organ sources. Acute thrombovascular rejection (AVR) is the main hurdle in xenotransplantation. Disparities in nucleotide metabolism in the vessels of different species may contribute significantly to the microvascular component of AVR.

**Methods:**

We evaluated the extent of nucleotide metabolism mismatch in selected organs and endothelial cells of different mammals with particular focus on the changes in activity of ecto-5’-nucleotidase (E5’N) elicited by exposure of porcine hearts or endothelial cells to human blood (ex vivo) or human plasma (in vitro).

**Results:**

E5’N activity in the rat heart was significantly higher than in other species. We noted a significant difference (*p*<0.001) in E5’N activity between human and pig endothelial cell lines. Initial pig aortic endothelial E5’N activity decreased in vitro after a three-hour exposure to human and porcine plasma while remaining constant in controls. Ex vivo perfusion with fresh human blood for four hours resulted in a significant decrease of E5’N activity in both wild type and transgenic pig hearts overexpressing human decay accelerating factor (*p*<0.001).

**Conclusions:**

This study provides evidence that mismatches in basal mammalian metabolic pathways and humoral immunity interact in a xenogeneic environment. Understanding the role of nucleotide metabolism and signalling in xenotransplantation may identify new targets for genetic modifications and may lead to the development of new therapies extending graft survival.

Human organ failure is one of the main causes of death and disability.^1^ Transplantation remains the most effective treatment approach but its application is limited by the severe shortage of human organ donors.^2^ Transplantation of animal organs is one possible solution to overcome this problem.^3^ However, destructive immune and thrombotic mechanisms that are triggered following transplantation between species lead to the destruction of the transplanted organ and must still be addressed.^3–5^ Although fundamental biological processes are generally similar across closely related mammalian species, several immunological incompatibilities and key metabolic differences exist that may lead ultimately to xenograft rejection.

Vascularised xenotransplants are subject to hyperacute rejection reactions resulting in an immediate organ failure.^6,7^ In the setting of pig-to-primate xenotransplantation, preformed xenoreactive antibodies have been implicated as key elicitors of immunological responses leading to hyperacute rejection. These antibodies recognise and bind to the galactose-α-1,3-galactose epitope present on the surface of pig endothelial cells,^8,9^ which in turn leads to activation of the complement system and destruction of graft cells.

Consistent with this model, the first successful approaches to overcome hyperacute rejection were directed towards inhibition of the complement cascade by expression of human complement regulatory molecules such as the human decay accelerating factor (hDAF) on the surface of endothelial cells from the donor organism.^10–12^ This strategy resulted in an unprecedented extension of organ survival from minutes to days or weeks in pig-to-primate transplants.^13–16^ More recently, increased attenuation of hyperacute rejection has been achieved using porcine α-1,3-galactosyltransferase knockouts, thereby preventing formation of the galactosea-1,3-galactose bond in polysaccharides present at the endothelial cell surface of donor organs.^17–19^ Following these spectacular advancements in effectively reducing hyperacute rejection, acute vascular rejection, causing xenograft destruction over a period of days to weeks, is now considered to be a major impediment.^7,20^

Particular interest has been drawn to nucleotide metabolism following the observation of remarkable differences between species such as a decrease in activity of ecto-5’nucleotidase (E5’N) by one order of magnitude in pigs relative to humans. Comparison of pig and human concentrations of nucleotide metabolites highlights other significant differences.^21^ Disparities in nucleotide metabolism between species may be important in the vascular component of acute humoural rejection^22,23^ and may be a major impediment to the application of xenotransplantation.^24,25^

Metabolic sequelae following humoural immune responses remain ill defined in transplantation. Nucleosides and their catabolites are known to inhibit activated mammalian immune cells although the extent to which these anti-inflammatory effects may be beneficial and have a potential implication for transplantation is unknown.^26^ Purine and pyrimidine precursors have crucial roles in determining nucleotide levels important for the maintenance of organ function, underscoring the need to understand the mechanisms of nucleoside metabolism and the implications in different animal models.

As the endothelium has been identified as the primary rejection target,^4^ we have focused on the importance of specific biochemical species’ differences in endothelial cells and their implications for acute vascular rejection in transplantation. We have also established other processes that are grossly different between species.^27,28^ Here, we evaluate and highlight some of these differences in different organs and cell lines from different mammals that are pertinent experimental models in the context of xenotransplantation research. Identification of these differences may lead to improvements of protection of endothelium during xenotransplantation or allotransplantation.

## Methods

Reagents for enzyme analyses and high performance liquid chromatography (HPLC) grade reagents were obtained from Merck (Poole, UK). [8-^14^C]Adenosine was obtained from ICN Pharmaceuticals (Basingstoke, UK). All other reagents were purchased from Sigma (Gillingham, Dorset, UK).

### Rodents

Male Lewis rats (14–16 weeks old; 210–310g [260.4 ±7.57g], *n*=5; Charles River, Margate, UK) and male C57BL mice (12– 14 weeks old; 24–32g [28.4 ±3.43g], *n*=5; Charles River) were bled after isoflurane anaesthesia (0.05ml unheparinised, non-citrated plastic Eppendorf tubes (Stevenage, UK), 0.5ml 1.3M perchloric acid). The tubes were placed immediately in liquid nitrogen. The chest cavities were opened and hearts dissected. The hearts were explanted, then rapidly freeze-clamped and stored in liquid nitrogen until analysis.

### Pigs

Wild type pigs (*n*=6) and transgenic pigs (*n*=5, *Sus* *scrofa*) constitutively overexpressing hDAF served as cardiac xenograft donors (male and female, age: 3–8 weeks, weight: 6.1–11.7kg). Heart tissue punch biopsies (1g) were taken from the left and right ventricles. All biopsies were placed immediately into liquid nitrogen for analysis. Venous blood samples were placed in plastic, unheparinised, non-citrated Eppendorf tubes (2ml) and frozen immediately in liquid nitrogen. Wedge biopsies of kidneys were taken for enzyme analysis.

### Baboons

Adult olive baboons (*Papio* *anubis*; *n*=3) weighing 9.9–16.8kg, supplied by the Southwest Regional Primate Research Center (San Antonio, TX, US), served as heart recipients. Prior to receiving xenotransplants or any test regimen, punch tissue biopsies (1g) were taken in duplicate from the left ventricle of the anaesthetised animal and placed immediately into liquid nitrogen. Baboon tissue was harvested at the time of cardiac xenograft or per protocol death (unpublished data).

### Humans

Whole human venous blood was collected from fasting donors (*n*=5). Human heart tissue samples (*n*=3), unacceptable for human transplant, were taken from beating heart donors whose ejection fraction was >40% (assessed by transoesophageal echocardiography). Right and left ventricular punch biopsies (in triplicate) were taken from the donor hearts and snap frozen in liquid nitrogen. Additionally, wedge tissue biopsies were performed on human kidneys not used for transplant (*n*=3). The biopsies were treated as above and stored in liquid nitrogen.

### Heart perfusion system

The perfusion system has been described previously^29^ and included the following components: a centrifugal pump (Medtronic, Watford, UK); a reservoir; a thermostatically controlled blood oxygenator (outflow connected to aortic cannula); a water jacketed heart compartment; a haemofilter; and a circulator maintaining the temperature at 37ºC. Wild type and transgenic pigs of either sex were anaesthetised and intubated. The chest was opened, and the aorta cannulated and clamped. Hearts were infused with cold cardioplegic solution before removal and connection to the perfusion apparatus. The system was filled with freshly collected heparinised human blood pooled from three ABO matched donors. Initial flow was adjusted to a perfusion pressure of 60mmHg (and maintained for 4 hours at 0.4–0.6l/min). Biopsy specimens were collected at the beginning and at the end of perfusion and specimens stored immediately in liquid nitrogen.

### Culture of endothelial cells

Pig aortic endothelial cells (PAEC) were prepared as described previously.^30^ Human umbilical vein endothelial cells, human aortic endothelial cells, PAEC and pig umbilical vein endothelial cells were obtained and cultured as described in detail previously.^31^

### Enzyme assays

The nucleotide metabolising enzymes were assayed in homogenates of heart biopsies collected before and at the end of perfusion as described previously.^32^ Porcine and human endothelial cells homogenates for enzyme assay were prepared from confluent endothelial cells.^32^ Erythro-9-(2-hydroxy-3-nonyl)adenine was used as an adenosine deaminase inhibitor. Each sample was analysed by reverse phase HPLC.^33^ Protein concentration was determined using the Bradford method.^34^ Activity of lactate dehydrogenase isozymes was measured as described by Wilson *et al*.^35^

**Figure 1:**
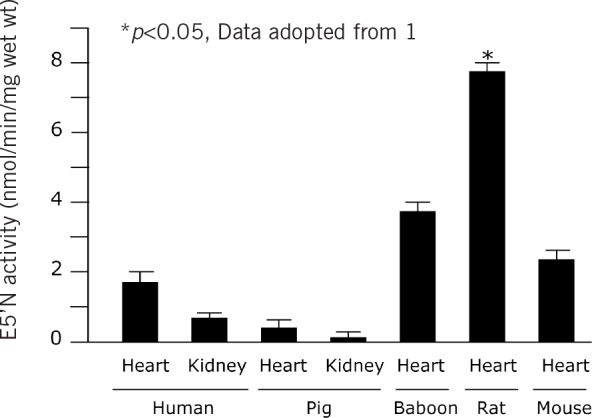
Mean ecto-5’-nucleotidase (E5’N) activity in species and organs relevant to xenotransplantation. Data adopted from 1.

### Blood purine and pyrimidine precursor and catabolite concentrations

Venous whole blood samples (0.5–2ml) were taken from mice (*n*=5), rats (*n*=5), pigs (*n*=11), baboons (*n*=3) and humans (*n*=3). Venous blood samples were collected prior to administration of any immunosuppressive or anticoagulation regimens. HPLC was used to evaluate levels of purines and pyrimidines as described previously.^33^

### Tissues homogenisation and enzymes analysis

Enzyme analysis in heart homogenates was performed as described in detail previously.^32^ The conversion of substrates into products was analysed by reverse phase HPLC. Protein content was determined by using the Bradford method.

### Exposure of endothelial cells to human and porcine plasma with and without complement inhibitor

Human and porcine blood was collected. Tubes were centrifuged immediately and the plasma was collected. One portion was heat inactivated at 56ºC for one hour. PAEC grown to confluence were washed in serum-free M199 media and incubated in media with 50% fresh human plasma (NHP), 50% heat inactivated human plasma (HHP) or 50% porcine plasma (PP). PAEC were incubated with human or porcine plasma for 0 or 180 minutes at 37ºC (0 minutes reflects that plasma was added to the cell homogenates and removed immediately and analysed). In another set of experiments, human C1 esterase inhibitor (CSL Behring, Haywards Heath, UK) was incubated with porcine and human treated plasma groups for up to 180 minutes at concentrations of 4mg/ml and 8mg/ml (data not shown). No significant changes in activity from control groups were seen. E5’N and other enzymes of nucleotide metabolism were assessed in cell homogenates as indicated above.

**Figure 2:**
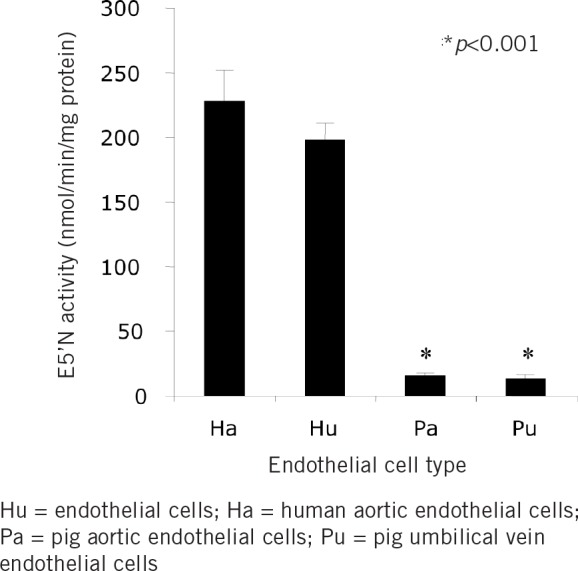
Mean ecto-5’-nucleotidase (E5’N) activity in human and porcine equivalent lysed endothelial cell types

### Statistical analysis

All values are presented as the mean ± standard error of the mean. In vitro cultured cells experiments were repeated 4–6 times (in triplicate each time), each controlled individually. Comparison of measurements was performed using the paired Student’s t-test. Comparison between groups was performed using the unpaired Student’s t-test or one-way analysis of variance (ANOVA). For analysis of enzyme activity during ex vivo perfusion, a non-parametric Mann–Whitney U test was used. Statistical significance (two-tailed) was defined as *p*<0.05.

## Results

### Species comparison of enzymes of nucleotide metabolism

E5’N activity in the rat heart was significantly higher than in the heart other species (*p*<0.005) as well as human and porcine kidneys. The rank order of E5’N activity was Rat > Baboon > Mouse > Human > Pig (*p*<0.05; Fig 1). A significant difference (*p*<0.001) was also noted in E5’N activity between human and pig endothelial cell lines (Fig 2).

Activity of other nucleotide metabolism enzymes across the different species and organs tested is shown in Table 1. The highest adenosine deaminase activity was found in the baboon heart (Baboon heart > Rat heart > Pig heart > Human heart > Mouse heart). Adenosine monophosphate deaminase activity was significantly different between species (Mouse > Rat > Human > Pig > Baboon; *p*<0.05) as was purine nucleotide phosphorylase and adenosine kinase activity (Mouse > Rat > Human > Baboon > Pig; *p*<0.05).

**Table 1:** Mean adenosine deaminase (ADA), adenosine monophosphate deaminase (AMPD), purine nucleotide phosphorylase (PNP) and adenosine kinase (AK)

Species	Organs	Enzyme activity (nmol/min/mg/wet tissue)			
		ADA	AMPD	PNP	AK
Human	Heart	0.73 ±0.63	1.64 ±0.17	1.23 ±0.09	0.15 ±0.02
Human	Kidney	0.43 ±0.07	1.42 ±0.11	3.52 ±0.48	
Pig	Heart	0.91 ±0.03	0.21 ±0.01	0.70 ±0.19	0.03 ±0.00
Pig	Kidney	0.14 ±0.02	1.11 ±0.10	12.30 ±0.50	
Baboon	Heart	4.34 ±0.07	0.16 ±0.04	2.99 ±0.71	0.05 ±0.00
Rat	Heart	1.62 ±0.23	3.77 ±0.19	1.45 ±0.25	0.20 ±0.01
Mouse	Heart	0.46 ±0.03	6.04 ±0.30	13.54 ±1.18	0.32 ±0.02

**Table 2:** Mean basal uric acid, purine and pyrimidine metabolite concentrations in mammalian whole venous blood samples

Species	Metabolites (nmol/ml)					
	Hypoxanthine	Inosine	Adenine	Uridine	Cytidine	Uric acid
Human	0.95 ±0.08	0.10 ±0.10	0.07 ±0.02	3.80 ±0.30	0.49 ±0.01	290.00 ±16.00*
Baboon	4.00 ±0.43	0.04 ±0.00	0.23 ±0.03	7.96 ±0.68	0.06 ±0.01	0.49 ±0.20
Pig	17.70 ±1.50	6.45 ±1.42	3.95 ±0.14	1.41 ±0.13	–	15.0 ±1.90
Mouse	0.67 ±0.21	0.86 ±0.41	1.52 ±0.33	38.40 ±5.37	2.55 ±0.05	11.32 ±1.06

* *p*<0.05

### Species-specific precursors and metabolites of purines and pyrimidines

Table 2 presents the results from the analysis of basal levels of nucleotide precursors and metabolites in the venous blood of different species. Notably, we found a higher baseline uric acid concentration in the venous blood of humans compared with the venous blood of pigs, mice and baboons (*p*<0.05), with the latter displaying the lowest level. Other differences were noted as shown in Table 2.

### Decreased E5’N activity on exposure to human plasma in vitro

**Figure 3:**
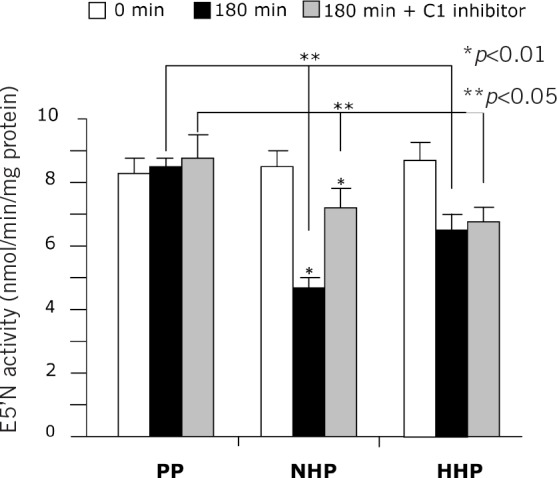
Mean ecto-5’-nucleotidase (E5’N) activity in pig aortic endothelial cell homogenates. Homogenates were incubated for 0 minutes or 180 minutes with 50% control porcine plasma (PP), 50% normal human plasma (nHP) or 50% heat inactivated human plasma (HHP) with or without human c1 inhibitor at 8mg/ml.

The initial pig aortic endothelial E5’N activity (9.15 ±1.87nmol/min/mg, 9.62 ±1.56nmol/min/mg and 9.10 ±1.40nmol/min/mg protein in NHP, HHP and control PP groups respectively) showed significant decrease after a three-hour exposure to human and porcine plasmas (4.58 ±0.47nmol/min/mg and 6.76 ±0.57nmol/min/mg protein in NHP and HHP groups respectively) while it remained constant at 9.62 ±0.88nmol/min/mg protein in PP controls (Fig 3). Incubation with C1 inhibitor added to NHP showed partial attenuation of decrease in E5’N activity, to the same level as in HHP. Separate experiments indicated that E5’N activity remained constant during three-hour incubation with Hanks’ balanced salt solution (results not shown).

### Decreased E5’N activity on ex vivo exposure of human plasma to transgenic pig heart

**Figure 4:**
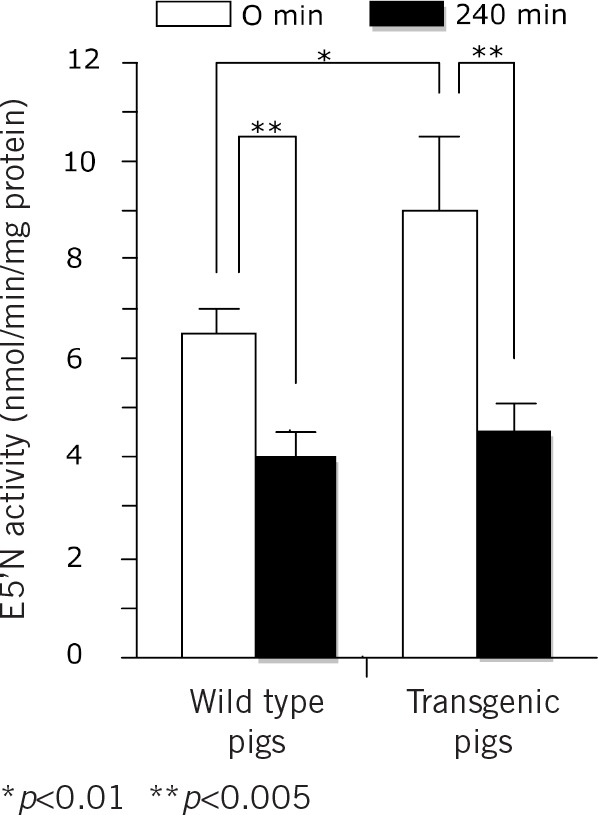
Mean effects of a four-hour perfusion of wild type or transgenic pig hearts overexpressing human decay accelerating factor with fresh human blood on ecto-5’-nucleotidase (E5’N) activity

E5’N activity in transgenic pig hearts overexpressing hDAF (8.54 ±2.10nmol/min/mg protein; *n*=5) was significantly higher than in the wild type pig hearts (6.60 ±0.33nmol/min/mg protein; *n*=6) at the start of perfusion with fresh human blood (*p*<0.01; Fig 4). Ex vivo perfusion of pig hearts with fresh human blood for four hours resulted in significant decrease of E5’N activity in both wild type (4.01 ±0.32nmol/min/mg protein) and transgenic pig hearts (4.52 ±0.52nmol/min/mg protein) (*p*<0.001). This decrease in E5’N activity occurred despite attenuation of immune, functional and structural changes typical of hyperacute rejection in transgenic hearts.

## Discussion

In this study we have demonstrated that there are significant differences in nucleotide metabolite levels and enzymatic activity between species relevant to transplantation. These novel data may provide further insights into the metabolic sequelae of acute vascular rejection in xenotransplantation and allotransplantation rejection mechanisms.

Additionally, we have demonstrated a potential mechanism of acute vascular rejection. This involves a reduction in the capacity of the endothelium to convert proinflammatory and proaggregatory extracellular nucleotides into adenosine due to a specific decrease in E5’N activity. The decrease in E5’N activity occurs rapidly following contact with human blood or plasma. Complement depletion (by heat inactivation) of human plasma or C1 inhibition partially attenuated E5’N activity decrease, while overexpression of hDAF in transgenic hearts was ineffective. Although controls with autologous porcine blood were not performed, our previous experience with purine enzyme activity measurements in a small animal perfusion system indicates that any non-specific effects are unlikely.^36^

## Conclusions

These results suggest that complement inhibition is not sufficient to fully protect porcine organs from injury on exposure to human plasma and highlights a role of additional factors in the process. Understanding these changes in E5’N activity in allotransplant and xenotransplant settings may lead to a better mechanistic understanding of acute vascular rejection, thromboregulation and xenograft cytoprotection.^37^
